# Enhancement of Adhesion Characteristics of Low-Density Polyethylene Using Atmospheric Plasma Initiated-Grafting of Polyethylene Glycol

**DOI:** 10.3390/polym13081309

**Published:** 2021-04-16

**Authors:** Taghreed Abdulhameed Al-Gunaid, Igor Krupa, Mabrouk Ouederni, Senthil Kumar Krishnamoorthy, Anton Popelka

**Affiliations:** 1 Center for Advanced Materials, Qatar University, P.O. Box 2713 Doha, Qatar; ta090196@qu.edu.qa (T.A.A.-G.); Igor.Krupa@qu.edu.qa (I.K.); 2 Materials Science and Technology Program, College of Arts and Science, Qatar University, P.O. Box 2713 Doha, Qatar; 3 Product Development & Innovation, Qatar Petrochemical Company (QAPCO), P.O. Box 756 Doha, Qatar; m.ouederni@qapco.com.qa (M.O.); s.krishnamoorthy@qapco.com.qa (S.K.K.)

**Keywords:** polyethylene, surface modification, corona discharge, polyethylene glycol, adhesion

## Abstract

The low-density polyethylene/aluminum (LDPE/Al) joint in Tetra Pak provides stability and strength to food packaging, ensures protection against outside moisture, and maintains the nutritional values and flavors of food without the need for additives in the food products. However, a poor adhesion of LDPE to Al, due to its non-polar surface, is a limiting factor and extra polymeric interlayers or surface treatment is required. Plasma-assisted grafting of the LDPE surface with different molecular weight compounds of polyethylene glycol (PEG) was used to improve LDPE/Al adhesion. It was found that this surface modification contributed to significantly improve the wettability of the LDPE surface, as was confirmed by contact angle measurements. The chemical composition changes after plasma treatment and modification process were observed by X-ray photoelectron spectroscopy (XPS) and Fourier transform infrared spectroscopy (FTIR). A surface morphology was analyzed by scanning electron microscopy (SEM) and atomic force microscopy (AFM). Adhesion characteristics of LDPE/Al adhesive joints were analyzed by the peel tests. The most significant adhesion improvement of the PEG modified LDPE surface was achieved using 10.0 wt.% aqueous (6000 M) PEG solution, while the peel resistance increased by approximately 54 times in comparison with untreated LDPE.

## 1. Introduction

Polyolefins are the largest class of synthetic thermoplastic polymers that are employed in a wide variety of applications nowadays, particularly food packaging, industrial applications, consumable products, structural plastics, and medical applications [1,2]. This is because these polymers are distinguished by light weight, excellent chemical and physical properties, cost effectiveness, as well as ease of processing [3,4]. Polyolefins are manufactured by cracking the petrochemical sources such as crude oil and natural gas [5,6]. Polyethylene (PE) polymers including low-density polyethylene (LDPE) and high-density polyethylene (HDPE) are the most famous polyolefins commonly employed in the food packaging industry, since they are easily heat sealable, can be fabricated into rigid films, with a good barrier against moisture and water vapor [7,8]. In spite of such features, their poor surface properties, including adhesion, wettability, and cytocompatibility, impede their integration with other materials to form multi-layered laminates. In fact, PE materials have low surface reactivity and hydrophobic nature due to the lack of functional groups and low proportion of polar regions on their surfaces, and therefore incomplete adhesion with other materials [9,10]. Therefore, several surface modification methods have been developed in recent decades. All these methods increase the surface energy of the polymer films, resulting in better wettability and thus higher bond strength [11]. The surface modification methods are basically classified into three sections: physical modification based on plasma technologies and flame treatment, chemical modification via surface functionalization, and mechanical abrasion [12,13,14]. However, previous studies found that mechanical abrasion could lead to significant damage of the treated surfaces [15]. The flame treatment is difficult to control, and bonding must be carried out shortly after exposure to flame [3]. Therefore, the use of plasma techniques and chemical methods are preferable in the surface treatment of polyolefins.

In the industrial scale, corona plasma discharge is a preferred cold plasma technique in surface modification of PE. It promotes surface activation, which leads to enhanced wetting and adhesion characteristics for applications related to adhesive bonding and printing [16,17]. Corona discharge is characterized by fast operation and completion (few seconds for treatment), cost effectiveness, easily adaptable to in-line operations, and environmentally friendly without the need to use aggressive chemicals during operation [3,18]. Corona discharge occurs when ambient air molecules are ionized at atmospheric pressure into charged particles such as electrons and ions. A high electric potential difference is formed between two asymmetric conductive electrodes (high-potential electrode and a grounded electrode) separated by a gap containing air. This creates a large electric field that accelerates the charged particles toward the polymer surface, which leads to the incorporation of reactive functional groups on the surface such as carbonyl, hydroxyl, hydroperoxides, aldehydes, ethers, esters, etc. The formed functional groups increase the polar part of surface energy and thus also the overall surface energy. Consequently, the surface is oxidized, its roughness and wettability increase, and finally its adhesion with other materials remarkably improves [11,17,19,20].

Chemical modification of the polymeric surfaces using graft polymerization plays a vital role in biomedical, environmental, and industrial applications [21,22], since it contributes to positive changes in the physical and chemical properties, morphology, and biocompatibility of the polymer [23,24,25]. Polyethylene glycol (PEG) is a versatile hydrophilic polyether that is immobilized onto the polymer surfaces using various techniques as physical adsorption, graft polymerization, covalent grafting, blending, etc. [26,27,28,29]. It is synthesized via chain-growth ring-opening polymerization of ethylene oxide in the presence of methanol or water as an initiator [30]. PEG is available as linear or branched chain polymers with an oxyethylene (-O-CH2-CH2-) repeating units that bonded with hydroxyl groups on either side of its chain [26,31,32]. The molecular weight of PEG plays a considerable role in specifying its properties. PEG is known as polyethylene oxide (PEO) when it is present in the form of a solid crystalline powder with molecular weight (M) greater than 20,000 g/mol, while PEG exists as viscous liquid (M < 1000 g/mol) or wax-like solid form (M: 1000–20000 g/mol) [26,33]. PEG and PEO compounds are soluble in both aqueous and organic solvents [34,35]. Recently, many studies focused on the development of the surface modification of hydrophobic polymers via graft polymerization with PEGs. Liu et al. worked on surface modification of polyester urethane (SPEU) films with different molecular weights of PEG compounds, Mn = 1200, 2400, and 4000 g/mol, for biomedical purposes. The SPEU surface was modified by grafting PEG on its surface, since PEG can effectively prevent protein adsorption and platelet adhesion due to its low interfacial free energy with water, unique solution properties, hydrophilicity, high chain mobility, and steric stabilization effect. The results showed that with increasing the molecular weight of PEG, there was a significant decrease in the water contact angle on PEG-g-SPEU, which indicated an increase in the surface energy and polarity, and thus strongly hydrophilic SPEU surface. Also, this can be attributed to high grafting density of PEG on the SPEU-PEG surface [36]. Adib and Raisi studied the surface modification of polyether sulfone membrane by grafting with hyperbranched PEG in combination with corona air plasma with the aim of enhancing anti-fouling properties. This led to improvement of the anti-fouling property and oil–water permeability of all modified membranes without any significant changes in oil rejection [37].

In this work, the plasma imitated grafting of PEG/PEO on the LDPE surfaces were employed to improve adhesion characteristics without changing bulk properties. In fact, this research shed light on studying the effect of the difference in the molecular weights of PEG/PEO as well as the concentration of the PEG/PEO-based prepared aqueous solutions in the enhancement of the surface properties of LDPE in order to achieve higher interfacial adhesion of modified LDPE with Al to form LDPE/Al adhesive laminates that are commonly used for food packaging and processing applications (e.g., Tetra Pak containers). However, evident changes in surface characteristics of LDPE specimens after PEG/PEO grafting were demonstrated using several analysis techniques. These include surface hydrophilicity or wettability, chemical compositions of the surface, as well as surface roughness and morphology. Last but not least, the adhesion strength between modified LDPE and Al was improved. The adhesion characteristics were analyzed using two different methods, namely peel test at constant 90° angle, and work of adhesion calculations based on contact angle measurements.

## 2. Materials and Methods

### 2.1. Materials

Low-density polyethylene (LDPE) from Qatar Petrochemical Company (QAPCO, Mesaieed, Qatar) with code number: EC01-049 was used in this research. The used LDPE in granular form were hot-pressed into a thin transparent sheet using a hydraulic press machine (Carver, Wabash, IN, USA). Some characteristics of the LDPE are summarized in Table 1. LDPE sheets were bonded with aluminum (Al) foil (GLAD^®^, Qingdao, Shandong, China) to produce a coherent adhesive joint (LDPE/Al laminates), achieving the main purpose of this work.

In addition, acetone (min.99.8% assay by G.C. method, Scharlab S.L., Barcelona, Spain) was used to remove any impurities or contaminants from the LDPE and Al surfaces prior to applying the surface treatment. For the wettability investigation of LDPE surfaces, ultra-pure water (Purity ≥ 99%, water purification system Direct-Q^®^, Millipore Corporation, Molsheim, France), formamide (Purity > 98%, FLUKA™, Merelbeke, Belgium), ethylene glycol (Purity ≥ 98%, FLUKA™, Morris Plains, NJ, Belgium) were used as testing liquids with different surface tension to determine the changes in surface total surface free energy and its components of the LDPE samples based on contact angle measurements. For surface modification of LDPE surfaces via grafting, PEG compounds with different molecular weights (M): 1000 g/mol (Fluka Chemika, Buchs, Switzerland), and 6000 g/mol (Merck KGaA, Darmstadt, Germany), as well as PEO with M = 300,000 g/mol (Sigma-Aldrich corporation, MO, St. Louis, USA) were used to increase the adhesion characteristics of LDPE surfaces. These compounds were dissolved into distilled water to prepare aqueous solutions at specific concentrations.

### 2.2. Preparation of LDPE Thin Sheets and LDPE-Al Laminate

The LDPE granulates were converted into coherent thin sheets using a hydraulic mounting press machine (Carver, Wabash, IN, USA). Ten grams of LDPE granules were placed between two transparent polyester sheets inside two highly polished stainless-steel plates, with a concern that granules were positioned adjacent to each other and on one level. After that, all the previously prepared were entered between the upper and lower molding plates of the hydraulic press machine. LDPE granules were heated up into a temperature slightly higher than the melting temperature (160 °C). Once the desired temperature was reached, a one-ton load was applied into the LDPE granules for two minutes, to convert these granules into a thin sheet under the influence of applied temperature and force. Finally, the prepared LDPE sheet was cooled down gradually until room temperature. The thickness of the LDPE sheets was found to be approximately 290 µm and LDPE samples were cleaned by acetone in order to remove all undesirable contaminants from the surface prior to every post treatment/modification process. Furthermore, the LDPE/Al adhesive joints were fabricated by lamination process using mounting hot press machine with almost the same steps as LDPE sheet preparation (two tons compression molding for 2 min at 160 °C, then cooling to room temperature).

### 2.3. Surface Modification of LDPE Surface Using Corona Discharge

**Figure 1:**
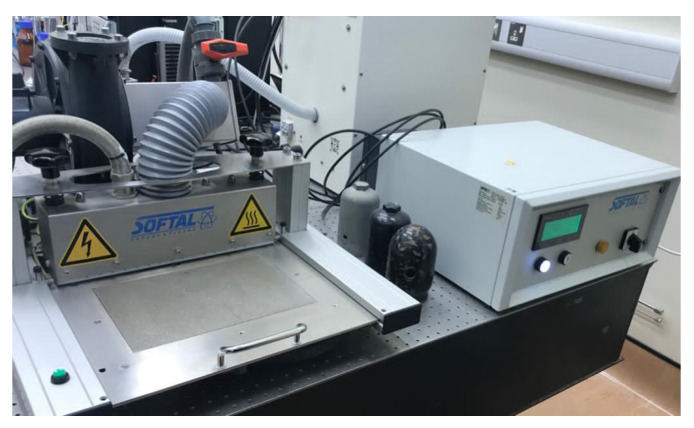
Corona plasma discharge system (Softal, Hamburg, Germany)**.**

The LDPE surface was treated using corona plasma discharge in order to introduction of polar functional groups. A laboratory scale corona plasma system (CVE-L, Softal, Hamburg, Germany) (Figure 1) was employed for surface treatment of LDPE foils under atmospheric pressure using 300 W of nominal power and 17.20 kHz of frequency. The plasma treatment process of LDPE was optimized by varying treatment time from 1 to 7 s, while the optimal treatment time was achieved using 5 s, which was associated with the best achieved wettability. This system contains a catalytic ozone removal system ensuring a safe working environment. Applied high potential between the biased and grounded electrode (1.5 mm gap distance) using ambient air was responsible for homogeneous surfaces treatment of LDPE. The LDPE samples were treated from both sides.

### 2.4. Grafting PEG/PEO onto LDPE

**Figure 2:**
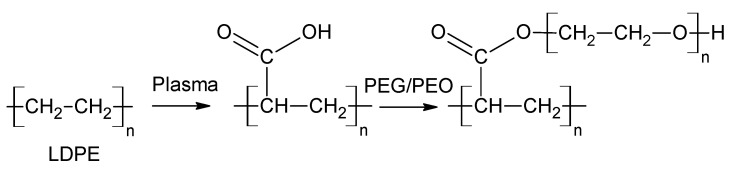
Scheme of proposed grafting mechanism of polyethylene glycol or polyethylene oxide (PEG/PEO) onto corona-treated LDPE.

The corona-treated LDPE specimens were completely immersed into specific concentrations of PEG- or PEO-based aqueous solutions at room temperature for 24 h (Figure 2). After the modification process, the LDPE specimen was thoroughly rinsed with distilled water immediately after extraction from the solution, to remove unreacted species from the LDPE surface. Thereafter, it was left to totally dry at room temperature prior to other characterizations and lamination with Al. Six different aqueous solutions were used in this work, regarding two different concentrations per each PEG/PEO molecular weight, to investigate the influence of changing the molecular weight of PEG/PEO chains, and concentration of prepared PEG/PEO aqueous solutions on the surface characteristics of LDPE. These concentrations were as follows: 1.5 wt.%, and 10.0 wt.% for 1000 M PEG, 1.5 wt.% and 10.0 wt.% for 6000 M PEG, and 1.5 wt.% and 5.0 wt.% (maximal solubility in water) for 300,000 M PEO.

### 2.5. Grafting Efficiency (GE) Evaluation

Grafting efficiency (GE) in the grafting process was defined as the percentage of the amount of the grafted monomer, which is linked into the polymer backbone to the total amount of the free polymer. GE values of the PEG/PEO grafted on LDPE (PEG/PEO-g-LDPE) specimens were calculated gravimetrically using Equation (1)

(1) $$\mathrm{GE}\;[\%]=\left(\frac{m_{1\;}-{\;m}_{0}}{m_{0}}\right)\times 100\%$$

where $m_{0}$ is the mass of the LDPE sample before grafting, $m_{1}$ is the mass of the LDPE sample after grafting with PEG/PEO.

### 2.6. Determination of Surface Wettability

The changes in surface wettability after plasma treatment and modification of the LDPE samples were investigated by measuring the contact angle of selected testing liquids. Three testing liquids with different surface tensions and polarities were employed in sessile drop contact angle measurements, such as water, formamide, and ethylene glycol (see Table 2). Contact angle measuring system OCA 35 (Dataphysics, Filderstadt, Germany) was used for this purpose. This system was connected to an optical video-base imaging system linked to high-resolution USB camera (up to 2200 images/s). According to the sessile drop method, 3 μL volume droplet of each testing liquid was deposed softly with constant dosing rate of 2 µL/s on the LDPE samples with the dimensions of 8 cm length × 2 cm width. Then, the contact angle was measured after 3 s to ensure that the liquid droplet spreads evenly and completely over the surface, while thermodynamic equilibrium was achieved. At least five separate readings for each testing liquid were taken to obtain one representative average contact angle value that was subsequently used in the calculation of solid/liquid interfacial tension based on the Owens-Wendt-Rabel-Kaelble method (OWRK-model). OWRK-model expresses the interfacial interactions along the solid and liquid molecules ($\gamma_{sl}$) in term of three components, the total surface free energy (γ) and its components: polar ($\gamma^{p}$) and dispersive ($\gamma^{d}$) components, by Equation (2).

(2) $$\gamma_{sl}=\gamma_{s}+\gamma_{l}-2\left(\sqrt{\left(\gamma_{s}^{d}.\gamma_{l}^{d}\right)}+\sqrt{\left(\gamma_{s}^{p}.\gamma_{l}^{p}\right)}\right)$$

### 2.7. Determination of the Adhesion Strength of LDPE/Al Laminate

The 90° peel test measurements were employed for the evaluation of the adhesion characteristics between LDPE and Al components that form together a coherent laminate. Peel tester LF-Plus (Lloyd Instruments, West Sussex, UK) based on ASTM D6862 standard test method was employed in the adhesion strength measurements. This system was connected to NEXYGENPlus testing software, which allows entering the basic data and experimental conditions that fit the test type, as well as the results displayed as numerical values and representative graphs. Laminated LDPE/Al strips with dimensions approximately of 8 cm height and 2 cm width were attached tightly on an acrylic two sided tape (3 M 4910 k, VHBTM) prior to starting the test. The peel strength (the force per unit width of the laminate) was measured under dynamic conditions: 1-kN load cell was applied at 90° angle peeling on the specimen, operated at slow speed rate (v = 10 mm/min) to ensure the applied peeling force is evenly distributed over the surface, and the test time was set at 360 s to ensure that LDPE ultra-thin layer was completely separated from the Al foil. The peel resistance (peel force per width) was evaluated from a 10–50 mm distance of the LDPE/Al laminate. Following the Standard Test Method for 90 Degree Peel Resistance of Adhesives (ASTM D6862); 4–5 separate readings of LDPE-Al adhesives were taken to acquire one average value of the peel resistance, and subsequently compared with the work of adhesion computed from contact angle measurements.

### 2.8. Calculation of the Work of Adhesion

**Figure 3:**
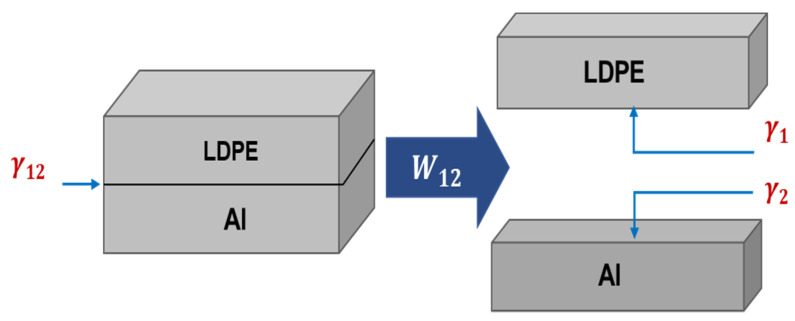
Illustrative scheme of work of adhesion between LDPE substrate and Al film.

The work of adhesion ($W_{12}$) for a solid–solid combination is defined as the reversible thermodynamic work (energy change per unit area) that is required to separate two adherent materials to form a laminate from the equilibrium state into a separation distance of infinity (Figure 3) [38]. In this work, quantities W_12_ of untreated, plasma treated and modified LDPE in the LDPE/Al laminate were calculated from contact angle measurements depending on the polarity and dispersion values of the surface energy by the Young–Dupré equation (Equation (3)), as follows [39]:

(3) $$W_{12}=\gamma_{1}+\gamma_{2}-\gamma_{12}$$

where $\gamma_{1}$ is the surface energy of LDPE, $\gamma_{2}$ is the surface energy of Al, $\gamma_{12}$ is the interfacial energy between LDPE and Al (solid–solid interface) and can be determined by the following equation.

(4) $$\gamma_{12}=\gamma_{1}+\gamma_{2}-2{\left(\gamma_{1}^{P}\times \gamma_{2}^{P}\right)}^{\frac{1}{2}}-2{\left(\gamma_{1}^{d}\times \gamma_{2}^{d}\right)}^{\frac{1}{2}}\;$$

by substituting Equation (4) into Equation (3); the work of adhesion ($W_{12}$) is obtained as follows:

(5) $$W_{12}=2[{\left(\gamma_{1}^{P}\times \gamma_{2}^{P}\right)}^{\frac{1}{2}}+{\left(\gamma_{1}^{d}\times \gamma_{2}^{d}\right)}^{\frac{1}{2}}]$$

where subscripts ‘1′ and ‘2′ refer to LDPE and Al respectively; the superscript ‘d’ represents to the non-polar/dispersive contribution; and the superscript ‘p’ refers to the polar contribution to the surface free energy.

### 2.9. Surface Morphology Analysis

The changes in two-dimensional surface morphology and roughness of the LDPE samples before and after surface modification were investigated by scanning electron microscopy (SEM) (Nova NanoSEM 450, Hillsboro, OR, USA). The LDPE specimens were observed at a high magnification (20,000×) and at high spatial resolution in order to achieve a high quality of the observed images. The working distance (WD) between the source of electrons and the exposed surface of the sample was set within the range of 4.6–5.1 mm. Furthermore, the SEM system was operated with moderate acceleration voltage equal to 5.0 kV. LDPE surfaces were coated by a thin layer (few angstroms thickness) of a gold (Au) to ensure higher resolution of captured SEM images, as well as to prevent charging of the surface and to promote the emission of secondary electrons [40].

The three-dimensional changes in the surface topography and roughness of the LDPE after plasma treatment and modification by PEG/PEO were determined using atomic force microscopy (AFM). The AFM images were obtained by an MFP-3D AFM device (Oxford Instruments Asylum Research, Abingdon, Oxford, UK) using AC160TS probe (Veeco model, OLTESPA, Olympus, Tokyo, Japan), which is covered with a thin reflex aluminum coating in order to prevent the light directed from the microscope lens towards the sample surface being scattered or lost. Furthermore, AFM measurements were conducted under ambient conditions in the dynamic mode in air (AC mode) known also as tapping mode. This mode is preferred due to it overcoming technical problems related with friction, adhesion, electrostatic forces that may appear after a plasma treatment and cause image data to be distorted [41]. Moreover, AFM is an ideal tool to quantitatively measure the dimensional surface roughness in nano-scale and to visualize the surface nano-texture of the deposited film, via commonly parameter that describe the vertical dimensions of the surface, namely average surface roughness line (Ra). Ra is defined as an arithmetical mean height of a line of the irregularities in the direction perpendicular to the sample surface [42,43].

### 2.10. Surface Composition Evaluation

Fourier-transform infrared spectroscopy (FTIR) was employed to identify the changes in chemical composition of the LDPE samples after plasma treatment and modification process. FTIR spectra were recorded using (Spectrum 400, PerkinElmer, Waltham, MA, USA) equipped with a ZnSe crystal allowing the analysis of data from 1.66 µm of the penetration depth. This FTIR spectra were captured within a wavenumber range of 500–4000 cm^−1^ at spectral resolution of 4 cm^−1^ in the absorbance mode to collect 8 scans with the aim to obtain accurate FTIR spectra.

The elemental and chemical compositions of the untreated, plasma-treated, and modified LDPE samples were evaluated using X-ray photoelectron spectroscopy (XPS) (Axis Ultra DLD, Kratos Analytical, Manchester, UK). XPS spectra were collected by irradiating a monoenergetic X-rays to the surface of a material, causing the emission of photoelectrons that are located within 10 nm from the underneath surface. Thus, the kinetic energy of the electrons emitted from each element present on the surface is analyzed, and the spectrum is obtained as a plot of the number of detected electrons per energy interval versus their kinetic energy. Furthermore, quantitative data were calculated based on the peaks formed by the individual elements according to the peak heights, areas, positions, and certain spectral features [44].

## 3. Results

### 3.1. Grafting Efficiency (GE)

**Figure 4:**
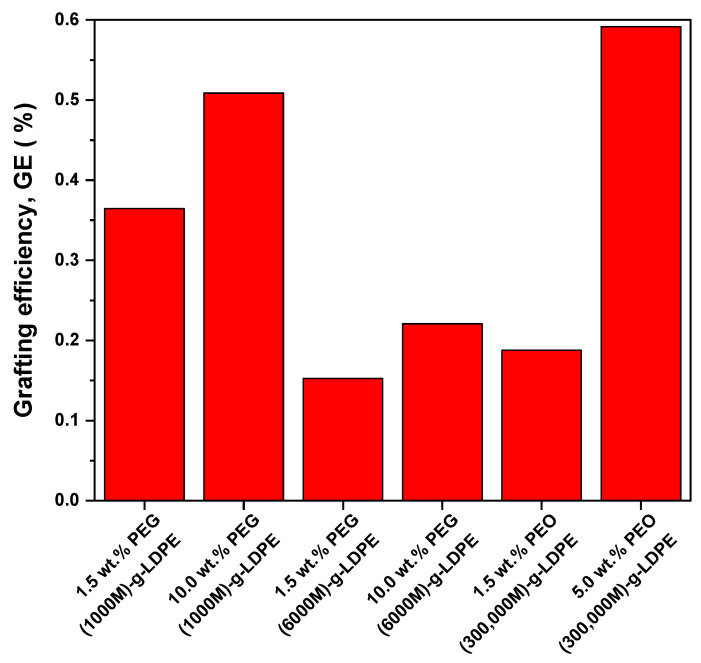
Effect of plasma treatment on grafting efficiency (GE) of PEG/PEO-g-LDPE.

The changes in the grafting efficiency (GE) of corona-treated LDPE surfaces modified by PEG/PEO are shown in Figure 4. The corona surface treatment had a significant effect on PEG/PEO grafting onto LDPE surfaces due to formation of radicals or reactive sites, which can react with PEG/PEO chains that are introduced into the surface. However, most probably, the grafting mechanism can be caused by an esterification process [45] as the result of interactions between the incorporated carboxylic groups in LDPE and hydroxyl groups of PEG/PEO [31] as was confirmed by FTIR measurements. This leads to an increase in the mass of the modified specimen, thus increasing GE [9]. It was noted that GE increased with increasing the PEG/PEO monomer concentration in the aqueous solution, due to incorporation of PEG/PEO chains onto LDPE surfaces. This was confirmed by the presence of the band at 1100 cm^−1^ (C-O-C) in the FTIR spectra [46]. Moreover, the highest GE was achieved for 5.0 wt.% PEO (300,000 M)-g-LDPE films preceded by 5 s of plasma surface treatment, while GE was approximately 0.6%. This can be explained by PEO (300,000 M) having ultrahigh molecular weights that can create a thicker layer on the LDPE surface in comparison to another PEG being used.

### 3.2. Surface Wettability Analysis

**Figure 5:**
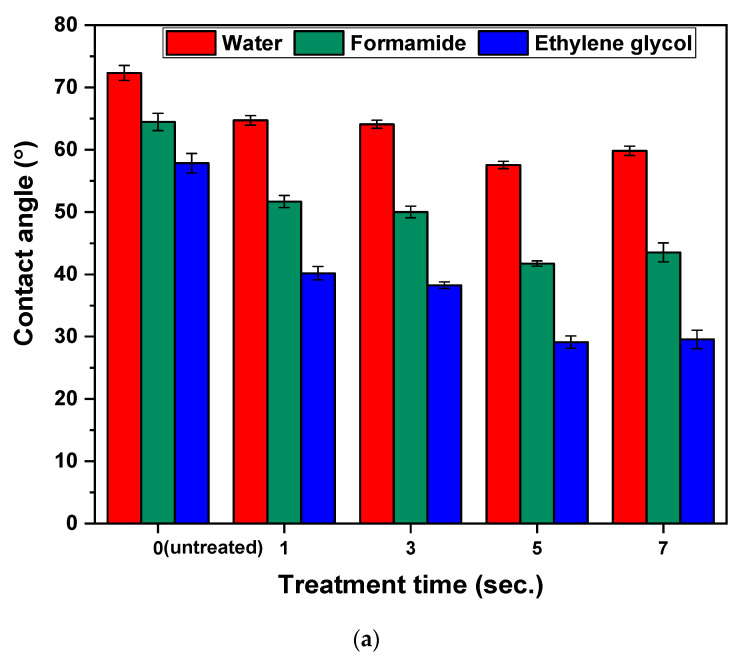

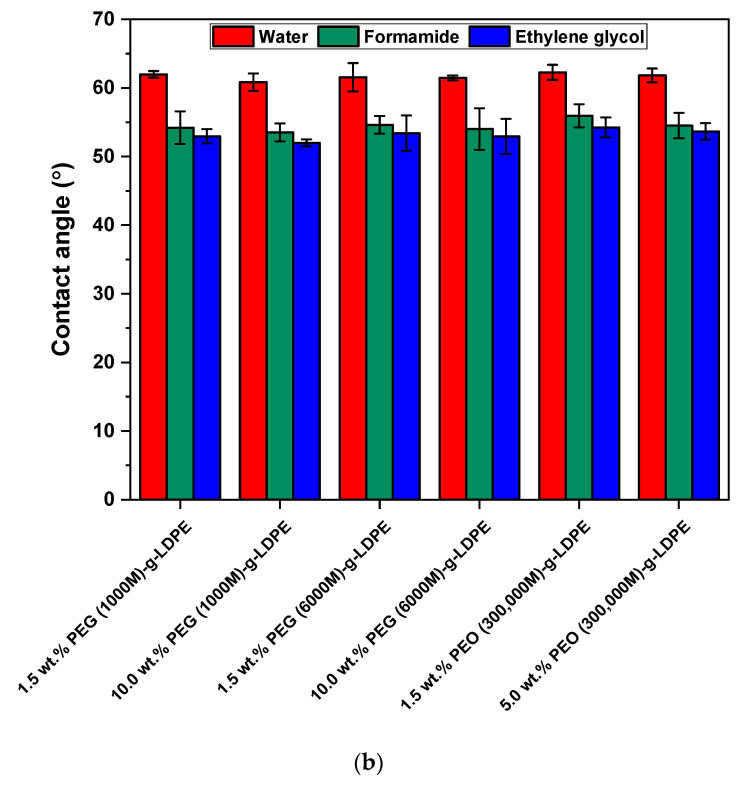
Contact angle of (**a**) LDPE samples vs. corona-treatment time, (**b**) PEG/PEO-g-LDPE.

The wettability property refers to the ability of a liquid to maintain in contact with a solid surface and it is characterized by the contact angle when a droplet of liquid is placed on a flat, horizontal solid surface. In this work, three testing liquids with various surface tension and polarity, namely water, formamide, and ethylene glycol, were used to study the changes in the wettability after plasma treatment and surface modification of LDPE. As can be seen from Figure 5a, a dramatic decrease in the contact angle values was observed with increasing the surface treatment time via corona discharge. The maximum decrease in the contact angle values was recorded after 5 s of surface treatment, so it can be considered as the optimum treatment time for LDPE surfaces. The contact angle values were decreased from 72.3° to 57.5° for water, from 64.5° to 41.7° for formamide, and from 57.8° to 29.1° for ethylene glycol corresponding to untreated and 5 s corona-treated LDPE surfaces. However, the relatively low values of contact angles of testing liquids for untreated LDPE were probably affected by the processing additives as was confirmed by pre-sent oxygen-containing groups observed by XPS. Plasma treatment leads to surface oxidation and introduction of new polar functional groups such as C=O, –OH, COOH, C–O–C, into the LDPE surfaces responsible for a wettability increase [47]. In addition, the effect of PEG/PEO grafting on the wettability of LDPE was studied (Figure 5b). A noticeable reduction in the contact angles of the PEG/PEO-g-LDPE surfaces were recorded compared to untreated LDPE surfaces. This can be explained by changes in surface roughness as a result of PEG/PEO grafting [48]. However, a slight increase was observed in the contact angle values for all the PEG/PEO-g-LDPE in comparison with only corona-treated LDPE, as a result of chemical nature of PEG/PEO. Furthermore, it was revealed that as the concentration of PEG/PEO aqueous solutions increased, the contact angle became slightly lower for the same molecular weight, due to enriching the modified surface with PEG/PEO grafted on the LDPE surface, as well as hydrophilic properties of the PEG/PEO molecules themselves [49].

**Figure 6:**
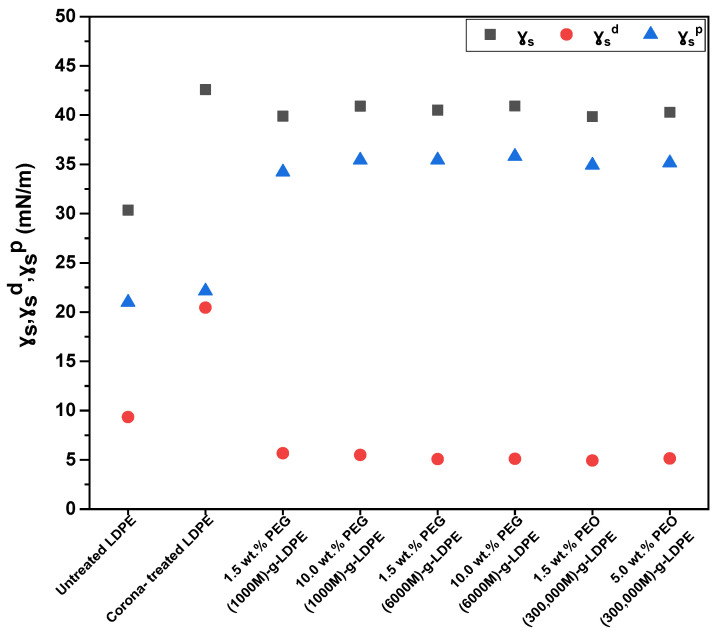
Surface energy ($\gamma_{s}$) and its components: polarity ($\gamma_{s}^{p}$ ), and dispersion ($\gamma_{s}^{d}$ ) of LDPE surfaces.

Figure 6 shows the change in the surface free energy and the corresponding polar and dispersive contributions of the untreated and modified LDPE samples. It became clear that the surface free energies were significantly increased after surface treatment with corona discharge from 30.3 mN/m for untreated LDPE to 42.6 mN/m for corona-treated LDPE due to introduction of characteristic polar functional groups, such as C=O, –OH, COOH, COO–, C–O–C, to the substrate surface [47]. It was confirmed that surface modification of LDPE via plasma-initiated grafting of PEG/PEO contributed to an improvement of the wettability properties of LDPE surfaces, as an increase in both the surface energies and polarities were observed for all PEG/PEO used with estimated percentages of 31.3% and 63.0%, respectively at the minimum. This indicated that the chemical character of grafted PEG/PEO affected on the surface hydrophilicity of LDPE substrate [50].

### 3.3. Surface Morphology Analysis

**Figure 7:**
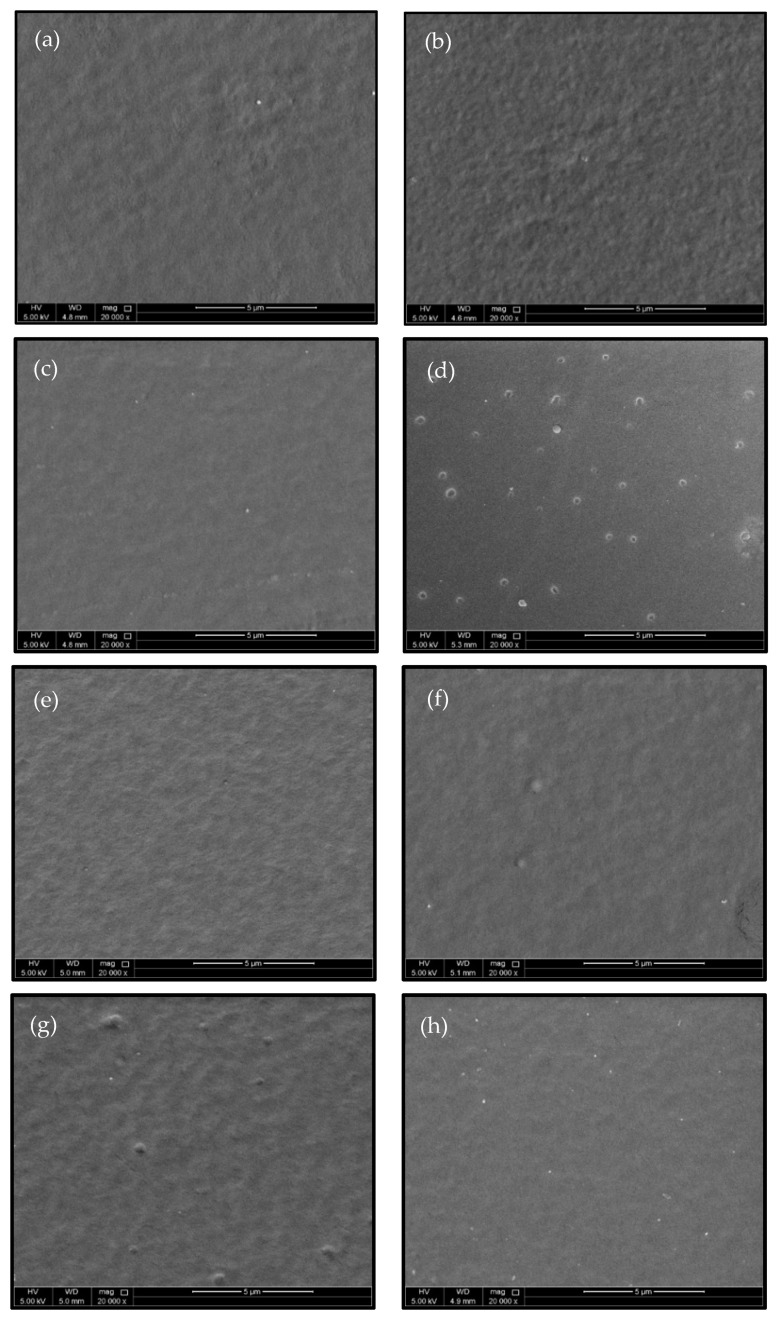
SEM micrographs of LDPE surface: (**a**) untreated, (**b**) corona treated, (**c**) 1.5 wt.% PEG (1000 M)-g-LDPE, (**d**) 10.0 wt.% PEG (1000 M)-g-LDPE, (**e**) 1.5 wt.% PEG (6000 M)-g-LDPE, (**f**) 10.0 wt.% PEG (6000 M)-g-LDPE, (**g**) 1.5 wt.%PEO (300,000 M)-g-LDPE, (**h**) 5.0 wt.% PEO (300,000 M)-g-LDPE.

The changes in the morphological characteristics of the corona-treated (5 s) and PEG/PEO grafted LDPE samples were investigated by SEM analysis, as shown in Figure 7. The SEM image of untreated LDPE surface (Figure 7a) showed it excelled at low levels of surface roughness, while a noticeable increase in surface roughness was observed to the corona-treated LDPE samples as a consequence of surface ablation and etching processes (Figure 7b). In contrast, a slight increase was observed in surface roughness predominantly in the amorphous phase of PEG/PEO-g-LDPE surfaces affected by 5 s of continuous treatment by corona discharge (Figure 7c–h). However, it can be seen that the surface roughness of the PEG/PEO-g-LDPE samples is lower compared to the only corona-treated LDPE surface due to the formation of a compact PEG/PEO layer.

**Figure 8:**
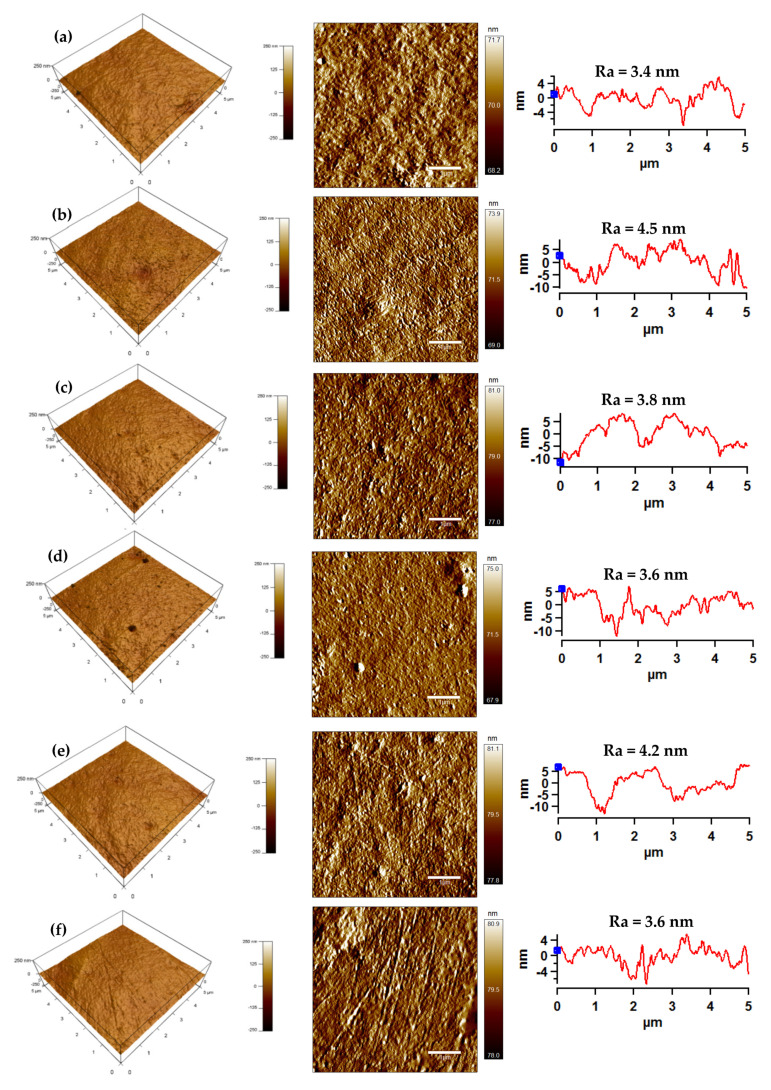

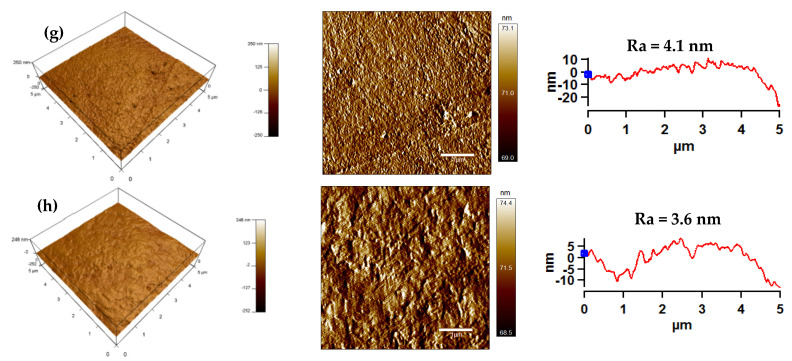
Atomic force microscopy (AFM) images (3D Height, Amplitude, line profile) with Ra roughness parameter of LDPE surface: (**a**) untreated, (**b**) corona-treated, (**c**) 1.5 wt.% PEG (1000 M)-g-LDPE, (**d**) 10.0 wt.% PEG (1000 M)-g-LDPE, (**e**) 1.5 wt.% PEG (6000 M)-g-LDPE, (**f**) 10.0 wt.% PEG (6000 M)-g-LDPE, (**g**) 1.5 wt.%PEO (300,000 M)-g-LDPE, (**h**) 5.0 wt.% PEO (300,000 M)-g-LDPE.

The AFM measurements were performed in order to analyze detailed surface morphology/topography changes in the LDPE surface after plasma treatment and modification processes (Figure 8). The changes in the surface roughness were quantified by the surface roughness parameter (Ra). AFM images showed that the surface of the untreated LDPE is relatively smooth with low value of average roughness (Ra = 3.4 nm), as demonstrated in Figure 8a. Correspondingly, the surface treatment of the LDPE surface with 5 s of corona treatment led to an increase in the surface roughness, while Ra increased to 4.5 nm as a result of etching and ablation processes (Figure 8b). The morphologies/topographies of the corona-treated grafted PEG/PEO LDPE specimens were detected by AFM analysis, as evidenced in Figure 8c–h. It was observed that surface roughness of the PEG/PEO-g-LDPE specimens were greater than the untreated LDPE, due to the PEG/PEO graft on the modified surfaces. These results are consistent with contact angle measurements, because rougher surfaces reduce hydrophobicity and thus improve the wettability characteristics [48]. It was noticed that surface roughness decreased after PEG/PEO grafting onto the LDPE surfaces compared to corona-treated surfaces, due to a formation of PEG layer onto the LDPE surface. Moreover, it was found that increasing the concentration of the PEG/PEO-based aqueous solution resulted in less rough LDPE surface, due to high grafting density of PEG/PEO that leads to the creation of a thin layer. Moreover, it was observed that all LDPE films grafted by high concentrations of PEG/PEO had the same surface roughness (Ra = 3.6 nm)

### 3.4. Chemical Composition Investigation

**Figure 9:**
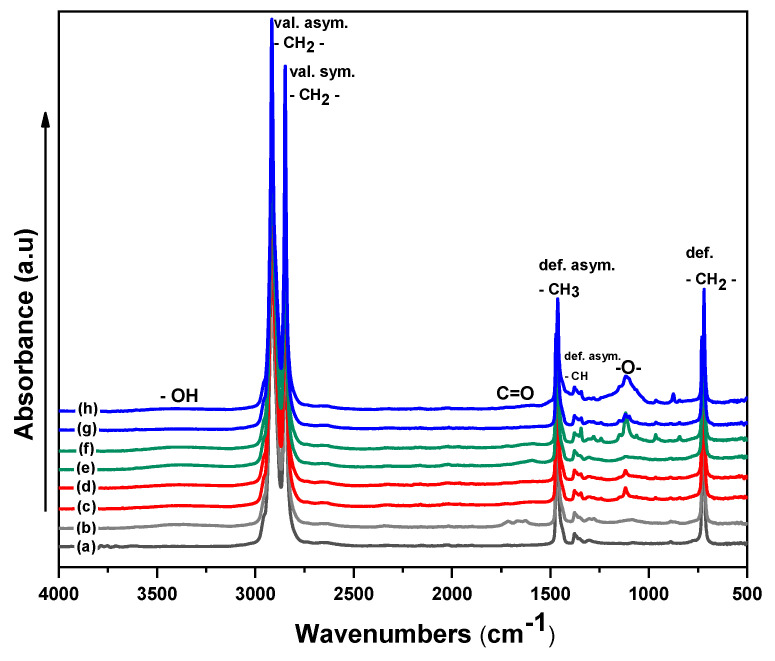
FTIR spectra of LDPE surfaces: (**a**) untreated, (**b**) corona-treated, (**c**) 1.5 wt.%PEG (1000 M)-g-LDPE, (**d**) 10.0 wt.%PEG (1000 M)-g-LDPE, (**e**) 1.5 wt.%PEG (6000 M)-g-LDPE, (**f**) 10.0 wt.%PEG (6000 M)-g-LDPE, (**g**) 1.5 wt.%PEO (300,000 M)-g-LDPE, (**h**) 5.0 wt.%PEO (300,000 M)-g-LDPE.

The FTIR analysis was used to identify the changes in the chemical composition of the LDPE surface after plasma treatment and surface modification by PEG/PEO (Figure 9). Generally, FTIR spectrum of untreated LDPE is characterized by characteristic absorption peaks, which coincide well with the relevant published literature, such as: out of phase and in-phase rock of the –CH2– at 720 cm^‒1^ and 731 cm^‒1^, weak asymmetric bending vibration of carbon-hydrogen bond (C–H) along the vertical axis (b-axis) of the LDPE chain at 1478 cm^−1^, asymmetric bending vibration of the CH3 groups along the horizontal axis (a-axis bend) at 1463 cm^−1^, as well as symmetric and asymmetric stretching vibrational bands that represent methylene group (C–H2) at 2848 cm^−1^ and 2916 cm^−1^, respectively [51]. The surface treatment by corona discharge led to significant appearance of new absorption bands at 1750 cm^−1^ and 1110 cm^−1^ associated with stretching vibrations of C=O (COOH) and –O– respectively. In addition, the hydroxyl functional group (–OH) was represented by a less intense and broad absorption peak between 3500 cm^−1^ and 3180 cm^−1^. The emergence of these oxygen-containing functional groups in the LDPE surfaces was caused by an oxidation process. In addition, the FTIR spectra of PEG/PEO-g-LDPE surfaces exhibited a noticeable increase in the peak intensity corresponding to –O– compared with only corona-treated LDPE samples, while the peak intensity of C–H decreased. Moreover, the FTIR spectra clearly indicated the disappearance of the COOH-associated absorption bands in the PEG/PEO-g-LDPE samples, which were utilized in the grafting process.

**Figure 10:**
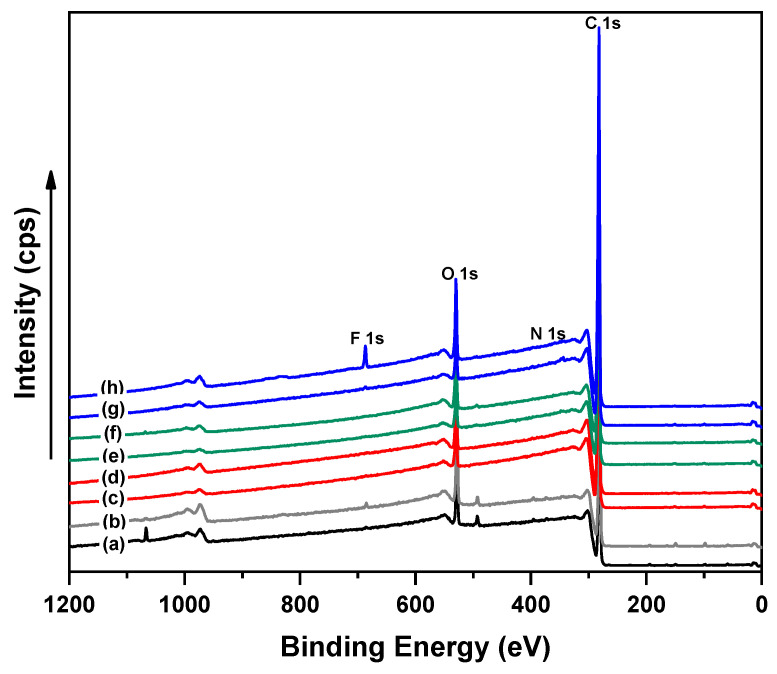
XPS spectra of LDPE surfaces.

The XPS technique provides quantitative information about the elemental compositions of the untreated, corona-treated, and modified LDPE surfaces as seen in Figure 10 and Table 3. As can be seen, there are two characteristic XPS peaks corresponding to the C1s and O1s at binding energy values of 284.8 and 532.8 eV, respectively. A slightly increase in the oxygen content was observed after corona treatment, while at.% of O1s increased from 8.6 to 11.3% for untreated and corona-treated LDPE, respectively, due to the enriching of the surface with oxygen-containing functional species. However, the presence of oxygen species in the untreated LDPE structure may be related to the processing additives. After PEG/PEO grafting onto LDPE surfaces via plasma treatment, it was found that the at.% of carbon element increased compared to untreated LDPE as results of higher carbon to oxygen ratio in PEG/PEO. Furthermore, a slight increase in the oxygen content was observed as the concentration of the PEG/PEO solutions increased and therefore at.% of carbon decreased, indicating higher density of PEG/PEO grafted on the LDPE surface. Furthermore, a decrease in the nitrogen content was observed on the LDPE surfaces with increasing the molecular weight of grafted PEG chains (>1000) due to a formation of a thin coating layer on the LDPE surface, which hinders the detection of the internal nitrogen element [36].

### 3.5. Adhesive Strength Measurements

**Figure 11:**
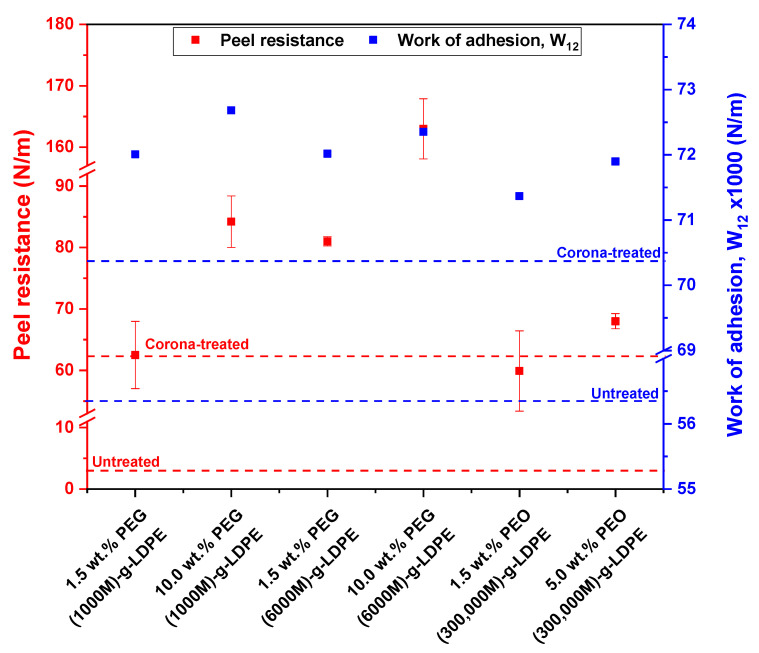
Effect of corona treatment on the peel resistance and work of adhesion of PEG/PEO-g-LDPE adhesive joint with Al.

The adhesive strength of the untreated, plasma treated and PEG/PEO-g-LDPE laminates with Al were analyzed using peeling resistance measurements, as shown in Figure 11. It can be seen that the peel resistance of the untreated LDPE/Al joints were nearly 3 N/m due to poor adhesion between the LDPE and Al laminate components. This can be interpreted to hydrophobic nature and low wettability of pristine LDPE surfaces. The peel resistance of LDPE/Al laminates remarkably increased (≈62.5 N/m) after plasma treatment using corona discharge as a result of improved wettability and roughness. In addition, it was observed that LDPE/Al adhesion joints prepared using the PEG/PEO-g-LDPE samples had a notable increase in peel resistance compared to untreated LDPE. This increase in peel resistance was mainly due to increase in the wettability and surface roughness caused by the incorporation of oxygen-rich functional. In addition, the highest peeling resistance values were recorded at high concentrations of PEG/PEO aqueous solutions, which was consistent with the surface wettability results obtained from the contact angle measurements of PEG/PEO-g-LDPE surfaces. The maximum peel resistance (163.0 N/m) of LDPE/Al adhesive joint was observed for LDPE/10.0% PEG (6000 M), which suggested as the optimum value. In contrast, the corona-treated LDPE grafted by PEO (300,000 M) surfaces exhibited the lowest adhesion values compared to other PEG used. This might be because as the molecular weight of PEG increased, the mole fraction of the reactive -OH groups decreased, to the point where the active bonding sites available on the LDPE surface are saturated and no more extend. Thus affecting on their surface hydrophilicity and thus led to reduced adherence to Al [52]. Moreover, work of adhesion ($W_{12}$) for the LDPE/Al adhesive joints were calculated using surface free energy and its components. It was found that $W_{12}$ values showed similar behavior as the peel resistance for all conditions used. The reason for the lower values of $W_{12}$ than peeling resistance of LDPE/Al adhesion joint was the fact that $W_{12}$ counts with infinite slow peeling rate, while crosshead speed during the peel resistance measurement was 10 mm/min. However, it was found that all modified PEG/PEO-g- LDPE surfaces had higher values of $W_{12}$ compared to untreated and corona-treated LDPE surfaces because of higher values of the polar component of the surface energy resulted from the improved wettability.

## 4. Conclusions

In this work, the surface characteristics of low-density polyethylene (LDPE) were enhanced using plasma-initiated grafting of different molecular weight polyethylene glycol or polyethylene oxide (PEG/PEO) onto LDPE surfaces in order to improve the adhesion to aluminum (Al) for industrial purposes. This surface modification improved the surface wettability as was confirmed by a decrease in the contact angles, and thus increased both the surface free energy and its polar component as a consequence of the change in the chemical composition of the modified LDPE surfaces. Moreover, the chemical composition analyses confirmed the presence of PEG/PEO on the corona-treated LDPE surface through esterification process. This led to considerable enhancement in the interfacial adhesion between LDPE and Al compared to the untreated and corona-treated surfaces. It was found that the adhesion strength between LDPE and Al surfaces were achieved at high concentrations of aqueous solutions containing PEG/PEO compounds. This could be due to improved wettability of the treated surfaces as confirmed by contact angle measurements as well as results obtained from typical surface analyzes. However, the highest adhesion in the LDPE/Al laminate was achieved by grafting with a 10% PEG (6000 M) aqueous solution onto 5 s corona-treated LDPE surface, where the peel resistance increased by approximately 54 times and 2.6 times compared to the peel resistance of untreated and corona-treated LDPE surfaces, respectively.

## Figures and Tables

**Table 1:** The properties/technical information of low-density polyethylene (LDPE) (EC01-049, QAPCO).

LDPE Properties	Description
Density at temperature 23 °C	0.918 g/cm^3^ (ASTM D-1505)
Melt flow index	8.0 g/10 min, 190 °C/2.16 kg (ASTM D-1238)
Crystalline melting point	105 °C
Recommended uses	Extrusion coating at high speed

**Table 2:** Surface free energy and its components: dispersion and polarity of testing liquids at 23 °C.

Testing Liquid	$\mathrm{Surface}\;\mathrm{Energy},\;\gamma_{l}(\mathrm{mN}/m)$	$\mathrm{Dispersion},\;\gamma_{l}^{d}(\mathrm{mN}/m)$	$\mathrm{Polarity},\;\gamma_{l}^{p}(\mathrm{mN}/m)$
Water	72.1	19.9	52.2
Formamide	56.9	23.5	33.4
Ethylene glycol	48.0	29.0	19.0

**Table 3:** Elemental composition of LDPE surfaces by XPS analysis.

Samples	Element, Atomic Conc. (at. %)		
	C 1s	O 1s	N 1s
(a) Untreated-LDPE	91.2	8.6	0.2
(b) Corona-treated LDPE	88.5	11.3	0.2
(c) 1.5 wt.% PEG (1000M)-g-LDPE	95.2	4.6	0.2
(d) 10.0 wt.% PEG (1000M)-g-LDPE	93.6	6.1	0.3
(e) 1.5 wt.% PEG (6000M)-g-LDPE	95.3	4.5	0.00
(f) 10.0 wt.% PEG (6000M)-g-LDPE	92.3	7.5	0.04
(g) 1.5 wt.% PEO (300,000M)-g-LDPE	95.1	5.2	0.03
(h) 5.0 wt.% PEO (300,000M)-g-LDPE	90.6	9.2	0.0
